# Vector-borne and other pathogens of potential relevance disseminated by relocated cats

**DOI:** 10.1186/s13071-022-05553-8

**Published:** 2022-11-08

**Authors:** Ricardo Guillermo Maggi, Vicky Halls, Friederike Krämer, Michael Lappin, Maria Grazia Pennisi, Andrew S. Peregrine, Xavier Roura, Bettina Schunack, Valeria Scorza, Séverine Tasker, Gad Baneth, Patrick Bourdeau, Dwight D. Bowman, Edward B. Breitschwerdt, Gioia Capelli, Luís Cardoso, Filipe Dantas-Torres, Gerhard Dobler, Lluís Ferrer, Luigi Gradoni, Peter Irwin, Frans Jongejan, Volkhard A. J. Kempf, Barbara Kohn, Susan Little, Maxime Madder, Carla Maia, Mary Marcondes, Guadalupe Miró, Torsten Naucke, Gaetano Oliva, Domenico Otranto, Barend L. Penzhorn, Martin Pfeffer, Ángel Sainz, SungShik Shin, Laia Solano-Gallego, Reinhard K. Straubinger, Rebecca Traub, Ian Wright

**Affiliations:** 1 Department of Clinical Sciences, North Carolina State University, Raleigh, NC USA; 2 International Cat Care, Tisbury, Wiltshire UK; 3 TransMIT GmbH, Giessen, Germany; 4 Department of Clinical Sciences, Colorado State University, Fort Collins, CO USA; 5 Department of Veterinary Sciences, University of Messina, Messina, Italy; 6 Department of Pathobiology, University of Guelph, Guelph, ON Canada; 7 Hospital Clínic Veterinari, Universitat Autònoma de Barcelona, Barcelona, Spain; 8 Bayer Animal Health, Elanco Animal Health Inc, Leverkusen, Germany; 9 Bristol Veterinary School, University of Bristol, Langford, UK; 10 Linnaeus Veterinary Limited, Shirley, UK; 11 Koret School of Veterinary Medicine, Hebrew University of Jerusalem, Rehovot, Israel; 12 Ecole Nationale Vétérinaire, Nantes, France; 13 Department Microbiology & Immunology, Cornell University, Ithaca, NY USA; 14 Istituto Zooprofilattico Sperimentale Delle Venezie, Legnaro, Italy; 15 Department of Veterinary Sciences, and Animal and Veterinary Research Centre (CECAV), University of Trás-Os-Montes E Alto Douro (UTAD), Vila Real, Portugal; 16 Aggeu Magalhães Institute, Fundação Oswaldo Cruz (Fiocruz), Recife, Brazil; 17 Bundeswehr Institute of Microbiology, Munich, Germany; 18 Department Animal Medicine and Surgery, Universitat Autònoma de Barcelona, Barcelona, Spain; 19 Istituto Superiore Di Sanità, Rome, Italy; 20 College of Veterinary Medicine, Murdoch University, Murdoch, WA Australia; 21 Department of Veterinary Tropical Diseases, University of Pretoria, Onderstepoort, South Africa; 22 Institute for Medical Microbiology and Infection Control and National Consiliary Laboratory for Bartonella (appointed by the Robert Koch Institute, Berlin, Germany), Goethe-University, Frankfurt Am Main, Germany; 23 Clinic of Small Animals, Freie Universität Berlin, Berlin, Germany; 24 Department of Pathobiology, Oklahoma State University, Stillwater, OK USA; 25 Clinglobal, Tamarin, Mauritius; 26 Global Health and Tropical Medicine, Instituto de Higiene e Medicina Tropical, Universidade NOVA de Lisboa, Lisbon, Portugal; 27 School of Veterinary Medicine, São Paulo State University, São Paulo, Brazil; 28 Animal Health Department, Veterinary Faculty, Facultad de Veterinaria, Universidad Complutense de Madrid, Madrid, Spain; 29 LABOKLIN GmbH, Bad Kissingen, Germany; 30 Department of Veterinary Medicine and Animal Production, University of Naples Federico II, Naples, Italy; 31 Department of Veterinary Medicine, University of Bari Aldo Moro, Bari, Italy; 32 Institute of Animal Hygiene and Veterinary Public Health, University of Leipzig, Leipzig, Germany; 33 College of Veterinary Medicine, Chonnam National University, Gwangju, South Korea; 34 Chair for Bacteriology and Mycology, Faculty for Veterinary Medicine, LMU Munich, Munich, Germany; 35 Melbourne Veterinary School, University of Melbourne, Parkville, VIC Australia; 36 The Mount Veterinary Practice, Fleetwood, Lancashire, UK

**Keywords:** Adoption, Animal welfare, Feline, Homing, Importation, Parasites, Bacteria, Viruses, Prevention, Relocation, Shelter, Zoonosis

## Abstract

Large populations of unowned cats constitute an animal welfare, ecological, societal and public health issue worldwide. Their relocation and homing are currently carried out in many parts of the world with the intention of relieving suffering and social problems, while contributing to ethical and humane population control in these cat populations. An understanding of an individual cat’s lifestyle and disease status by veterinary team professionals and those working with cat charities can help to prevent severe cat stress and the spread of feline pathogens, especially vector-borne pathogens, which can be overlooked in cats. In this article, we discuss the issue of relocation and homing of unowned cats from a global perspective. We also review zoonotic and non-zoonotic infectious agents of cats and give a list of practical recommendations for veterinary team professionals dealing with homing cats. Finally, we present a consensus statement consolidated at the 15th Symposium of the Companion Vector-Borne Diseases (CVBD) World Forum in 2020, ultimately to help veterinary team professionals understand the problem and the role they have in helping to prevent and manage vector-borne and other pathogens in relocated cats.

**Graphical Abstract:**

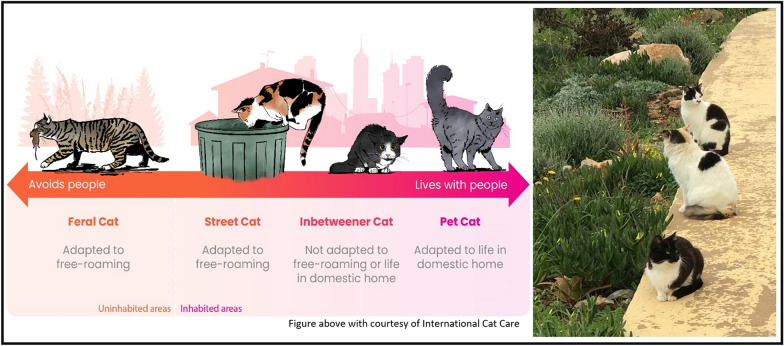

## Background

At the 15th Symposium of the Companion Vector-Borne Diseases (CVBD) World Forum in 2020, and subsequently at the International Society of Feline Medicine (ISFM) Symposium in 2021, the global and public health impact of the spread of vector-borne pathogens in relocated cats was discussed. Although this topic has received attention in dogs [1], it can be overlooked in cats. In this article, we discuss the issue of relocation and homing of unowned cats from a global perspective. We also review zoonotic and non-zoonotic infectious agents of cats and present a list of practical recommendations for veterinary team professionals dealing with homing cats. Finally, we present a consensus statement on this topic to help veterinary team professionals understand the problem and the role they have in helping to prevent and manage vector-borne and other pathogens in relocated cats.

## Relocation and homing of cats

Global pet cat populations are tracked by pet product industries, with the current world pet cat population exceeding 373 million cats [2]. Providing an equivalent estimate for unowned cats is more complex, but this number has been suggested to be as high as 600 million [3]. Intact free-roaming cats reproduce rapidly, and, in response, homing organizations and individuals look for ways to remove these cats from their current situation with the intention to relieve suffering and resolve complaints from the community. If strategic, well-planned and executed population management programs (i.e. high-volume TNR) existed throughout the world it is likely that many cat relocation decisions would not be required.

**Fig. 1:**
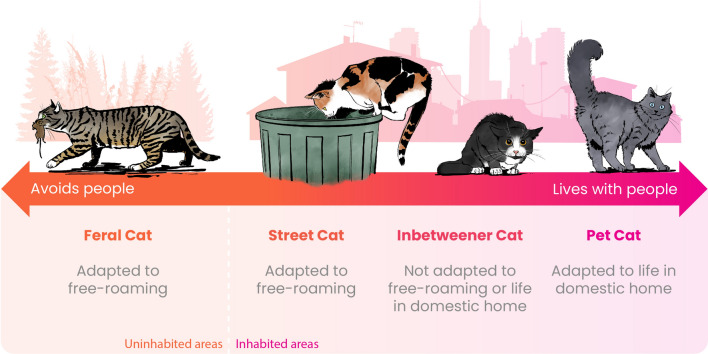
Categorization of cat lifestyles [6], which exist as a spectrum (figure with courtesy of International Cat Care)

The relocation and homing^1^ of cats are linked to economic, cultural and environmental factors. They are also very much linked to how populations of unowned cats are managed [4], which in turn is influenced by the lifestyle of the cats involved [5]. Globally, a spectrum of cat lifestyle categories exists, i.e. feral, street, inbetweener and pet cat (see Fig. 1) [6]. Categorization of a cat’s lifestyle depends on its desire to live with people and whether it can live independently of people or within a household.

Unowned cats from all categories can be found in homing centers, where they are housed awaiting adoption or taken into foster care. However, awareness of the needs of these cats is important for ensuring that homing, including relocation, is truly an appropriate outcome for these cats. Homing cats that are unsuitable to be kept as pets (particularly feral and street cats) can cause severe distress for these animals. Additionally, the stress involved in travel and relocation of cats, including those that are adapted to live in households as pets, is also important to consider; recent data from the UK suggest that increasing numbers of cats are imported from abroad to be pets [4]. Important disease implications also exist, such as importation of vector-borne pathogens, which are not native to the geographical relocation destination. Some of these infectious agents also have zoonotic potential [7].

Education of veterinary team professionals, as well as individuals engaged in relocation activities, in understanding the risks involved in inappropriate homing and relocation to different parts of the world and in how to recognize, diagnose, treat and prevent infections is important. One charity has outlined a possible approach to testing imported cats [8]. Additionally, online resources (https://cvbd.elanco.com; https://www.esccap.org; https://capcvet.org/; https://www.troccap.com/; http://www.abcdcatsvets.org/) and open access papers [9, 10], including descriptions of infection manifestations and maps documenting risks in different geographical areas, are freely available.

## Infectious agents to consider in homed cats

Many infectious disease agents carried by cats are zoonotic [7]. Viral, bacterial (including rickettsial), mycotic and parasitic agents are most common, with many of the bacterial and parasitic agents being vector-borne. A common misconception is that cats are less infested with vectors (such as ticks, mites, fleas) and are also less frequented by temporary vectors (mosquitoes, sand flies and flies) than other companion animals such as dogs. However, vectors and vector-borne pathogens, non-zoonotic as well as zoonotic, commonly occur in cats. For some feline infections, there are known significant differences in prevalence levels based on the type of cat. For example, infection of the domestic cat with the three main retroviruses, feline leukemia virus (FeLV), feline immunodeficiency virus (FIV) and feline foamy virus, is generally highest in cats allowed outdoors and in contact with other cats [11–13]. We herein provide in Table 1 a detailed listing of potentially occurring vector-borne pathogens and their corresponding vectors in cats. However, the relevance and relative risk for each pathogen may vary broadly, e.g. depending on geographical location of the homed cat.

**Table 1:** Feline vector-borne pathogens (including modes of transmission, potential clinical and laboratory findings and zoonotic potential) (*suspected in cats; **reported in other host species, but not yet in cats)

Genus	Species	Modes of transmission	Clinical and laboratory findings (most common findings in case of illness, generally often subclinical infection)	Zoonotic potential
Bacteria				
*Anaplasma*	*A. bovis*	Tick bites	Unknown	Unknown
	*A. phagocytophilum*	Tick bites	Fever, thrombocytopenia, suspected suppurative polyarthritis	Yes
	*A. platys*	Tick bites	suspected fever and thrombocytopenia	Yes
*Bartonella*	*B. clarridgeiae*	Flea feces Animal bites, scratches	Fever, lymphadenomegaly, suspected endocarditis	Yes
	*B. elizabethae*	Flea feces Animal bites, scratches	Suspected endocarditis	Yes
	*B. henselae*	Flea feces, tick bites** animal bites, scratches	Fever, lymphadenomegaly, endocarditis, myocarditis	Yes
	*B. koehlerae*	Flea feces Animal bites, scratches	Fever, lymphadenomegaly, suspected endocarditis	Yes
	*B. quintana*	Human body louse bites**, flea bites* Animal bites, scratches	Fever, endocarditis	Yes
	*B. vinsonii berkhoffii*	Tick bites** Animal bites*	Recurrent osteomyelitis	Yes
*Borrelia*	*B. burgdorferi*	Tick bites	Fever, lethargy, suspected suppurative polyarthritis	Yes
	*B. hispanica*	Tick bites, sucking lice bites	Weight loss, regenerative anemia, monocytosis	Yes
	*B. miyamotoi*	Tick bites, sucking lice bites	Unknown	Yes
	*B. persica*	Tick bites, sucking lice bites	Lethargy, anorexia, anemia, thrombocytopenia, icterus	Yes
*Coxiella*	*C. burnetii*	Flea bites*, tick bites* Direct contact*, fomites*	Abortion, stillbirth	Yes
*Ehrlichia*	*E. canis*	Tick bites	Fever, thrombocytopenia, lymphadenomegly	Yes
	*E. chaffeensis*	Tick bites	Few data available	Yes
	*E. ewingii*	Tick bites	Few data available	Yes
*Francisella*	*F. tularensis*	Tick bites**, blood sucking vectors (mechanical)** Animal bites*, ingestion**, scratches*	Fever, lymphadenomegaly, cough, dyspnea	Yes
Haemotropic *Mycoplasma* spp.	'*Candidatus* M. haemominutum'	Flea bites*, tick bites* Animal bites*, vertical**	Fever, hemolytic anemia	No
	'*Candidatus* M. turicensis'	Flea bites*, tick bites* Animal bites*, vertical**	Fever, hemolytic anemia	No
	*M. haemofelis*	Flea bites*, tick bites* Animal bites*, vertical**	Fever, hemolytic anemia	Yes
*Neorickettsia*	*N. mikurensis*	Tick bites**	Unknown; suspected lymphadenomegaly and fever	Yes
	*N. risticii*	Ingestion of infected trematodes**	Unknown; suspected anorexia, lymphadenomegaly and diarrhea	Unknown
*Rickettsia*	*R. conorii*	Tick bites	Suspected fever	Yes
	*R. felis*	Flea bites	Unknown; suspected fever	Yes
	*R. massiliae*	Tick bites	Suspected fever	Yes
	*R. rickettsii*	Tick bites	Suspected fever	Yes
	*R. typhi*	Flea bites	Suspected fever	Yes
*Yersinia*	*Y. pestis*	Flea bites direct contact, ingestion of infected host/prey	Fever, necrotic stomatitis, lymphadenomegaly, cough, dyspnea	Yes
Protozoa				
*Babesia*	*B. canis*	Tick bites** vertical**	Mild chronic anemia (few data available)	Unknown
	*B. felis*	Tick bites*	Hemolytic anemia, icterus, increased ALT	Unknown
	*B. lengau*	Tick bites*	Anemia (few data available)	Unknown
	*B. leo*	Tick bites*	Anemia (few data available)	Unknown
	*B. vogeli*	Tick bites**	Unknown	No
	*B. panickeri*	Tick bites*	Anorexia, lethargy, hemolytic anemia (few data available)	Unknown
	*B. hongkongensis*	Tick bites*	Unknown	Unknown
	*B. presentii*	Tick bites*	Anemia, fever, icterus, lethargy (few data available)	Unknown
*Cytauxzoon*	*Cytauxzoon felis*	Tick bites	Fever, icterus, splenomegaly, hepatomegaly, fatal infection	Unknown
	*Cytauxzoon* sp.	Tick bites*	Sub-clinical to rare severe fatal infection with anemia and fever	Unknown
*Hepatozoon*	*H. canis*	Ingestion of intermediate hosts (ticks)**, ingestion of paratenic hosts**, vertical**	Unknown	Unknown
	*H. felis*	Unknown, vertical*	Icterus, pancytopenia, increased SDMA (symmetric dimethylarginine)	Unknown
	*H. silvestris*	Unknown	Myocarditis	Unknown
*Leishmania*	*L. amazonensis*	Sand fly bites*	Skin and mucosal lesions (nodular, ulcerative)	Yes
	*L. braziliensis*	Sand fly bites*	Skin and mucosal lesions (nodular, ulcerative)	Yes
	*L. infantum*	Sand fly bites Animal bites**, blood transfusion**, vertical**	Skin and mucosal lesions (nodular, ulcerative), lymphadenopathy, ocular lesions (conjunctivitis, uveitis), hyperglobulinemia, anemia, proteinuria	Yes
	*L. mexicana* (*L. venezuelensis*)	Sand fly bites*	Skin and mucosal lesions (nodular, ulcerative)	Yes
*Trypanosoma*	*T. brucei (brucei)*	Tsetse fly bites, tabanids and other biting flies (mechanical)	Fever, emaciation, anorexia, edema and erythema (head, face), alopecia, lymphadenomegaly, ocular lesions (keratitis, conjunctivitis, photophobia, epiphora, pannus, hypopyon), eventual lethal outcome	Yes
	*T. congolense*	Tsetse fly bites, tabanids and other biting flies (mechanical)	Emaciation, anemia, no ocular signs, fatal outcome	Yes
	*T. cruzi*	Triatomine feces	Seizures, transient posterior paralysis (few data available)	Yes
	*T. evansi*	Tabanids and stable flies (mechanical), vampire bats bites**	Fever, weight loss, diarrhea and vomiting, edema (head, face), lymphadenomegaly, ocular lesions (corneal opacity), regenerative anemia, icterus, hindlimb instability, muscle atrophy, potential fatal outcome	Yes
Viruses				
Feline calicivirus (FCV)		Flea bites* direct contact, fomites	Fever, stomatitis, upper respiratory signs, polyarthritis, vasculitis with systemic variants	No
Dabie bandavirus (formerly: Severe Fever with Thrombocytopenia Syndrome Virus [SFTSV])		Tick bites Animal bites, direct contact, scratches	Fever, anorexia, lethargy, vomiting, thrombocytopenia, leukopenia	Yes
West Nile virus (WNV)		Mosquito bites	Neurologic signs	Yes
Helminths				
*Acanthocheilonema*	*A. dracunculoides*	Flea bites**	Unknown	Unknown
	*A. reconditum*	Flea bites**, lice (sucking and chewing) bites/ingestion**	Unknown	Unknown
*Aelurostrongylus*	*A. abstrusus*	Ingestion of intermediate hosts (slugs and snails), ingestion of paratenic hosts, ingestion of free larvae in snail/slug slime	Cough, dyspnea	No
*Brugia*	*B. malayi*	Mosquito bites	Lymphadenomegaly, lymphedema (few data available)	Yes
	*B. pahangi*	Mosquito bites	Lymphadenomegaly, lymphedema (few data available)	Yes
*Cercopithifilaria*	*C. grassii*	Tick bites	Unknown	Unknown
*Dirofilaria*	*D. immitis*	Mosquito bites	Cough, dyspnea, sudden death	Yes
	*D. repens*	Mosquito bites	Subcutaneous nodules, dermatitis	Yes
*Troglostrongylus*	*T. brevior*	Ingestion of intermediate hosts (slugs and snails), ingestion of paratenic hosts, ingestion of free larvae in snail/slug slime, vertical, transmammary	Cough, dyspnea, nasal discharge	No
	*T. subcrenatus*	Unknown	Cough, dyspnea, nasal discharge	No
*Thelazia*	*T. californiensis*	Deposition by secretophagous house flies (*Fannia canicularis*, *Fannia benjamini*)	Blepharospasm, epiphora, conjunctivitis, keratitis	Yes
	*T. callipaeda*	Deposition by secretophagous fruit flies (*Phortica variegata*, *Amiota okadai*)	Blepharospasm, epiphora, conjunctivitis, keratitis	Yes
*Dipylidium*	*D. caninum*	Ingestion of intermediate hosts (fleas, lice)	Unthrifty, rarely vomiting or diarrhea	Yes

### Non-zoonotic infectious agents

Most viral agents of cats are non-zoonotic (e.g., feline retroviruses; feline coronaviruses; feline herpesvirus-1 [FHV]; feline caliciviruses [FCV]). These agents are easily transmitted by direct contact with other cats, their secretions or vertical transmission. Thus, most feline viral infections are most common in populations of cats with direct contact. This is particularly true for the respiratory tract agents, FHV and FCV [14–17]. These agents can be carried by cats even if vaccinated, and stressful conditions such as homing can induce repeated shedding of FHV, potentially leading to infection of additional cats. Hypervirulent FCVs are one of the most dangerous examples of this problem as these variants can cause fatal infection even in adult cats previously vaccinated for other FCVs [14, 16]. While the feline retroviruses are non-zoonotic, if they cause immune deficiency, the risk of shedding of other infectious agents, including zoonotic ones, may increase [18, 19]. As the early stages of retroviral infections can be subclinical, testing for FeLV and FIV in all cats being homed is strongly recommended.

Among deep not zoonotic mycotic infections, *Cryptococcus* spp. and *Aspergillus* spp. can cause severe feline diseases, but they are non-contagious [20]. Cats with cryptococcosis are good sentinel species for environmental contamination and the consequent risk of exposure for humans and animals.

Feline *Mycoplasma* spp. commonly colonize mucus membranes of cats, and the respiratory agents in this group are occasionally associated with clinical disease and are contagious among cats and thus likely more common in cats housed together [21–23].

The type of cat is also potentially associated with increased prevalence rates for several non-zoonotic parasitic agents. For example, cats allowed outdoors are inclined to hunt and have increased risk of exposure to infectious agents carried by intermediate or paratenic hosts. Examples include the protozoan *Cystoisospora felis*, several cestodes such as *Taenia taeniaeformis* and the lungworms *Aelurostrongylus abstrusus* and *Troglostrongylus* spp. Most other common parasitic agents of cats are zoonotic and are listed below in the corresponding section. Periodic deworming is critical to avoiding environmental contamination with eggs and larvae of helminths. Cats exposed to the feces of other cats may also be more likely infected by other non-zoonotic protozoal agents such as *Tritrichomonas blagburni* (previously referred to as feline isolates of *Tritrichomonas foetus*). Again, other important protozoal agents in this context are zoonotic and are listed below.

Fleas, mosquitoes, sand flies and ticks are the common vectors for feline vector-borne pathogens. However, only a few of these are non-zoonotic or possess unknown zoonotic potential, of which the latter is represented by the protozoan genera *Babesia*, *Cytauxzoon* and *Hepatozoon* (see Table 1). While indoor cats are still at risk, cats with access outdoors are more commonly exposed to vectors in general, emphasizing the importance of vector control.

### Zoonotic infectious agents

Zoonoses are infections that are naturally transmitted between animals and humans, or between humans and animals [24]. Thus, in contrast to the terms ‘anthropozoonosis’ (transmission from animals to humans) and ‘zooanthroponosis’ (transmission from humans to animals) [25], the term zoonosis is non-directional and can refer to either route of transmission [24].

Even though there is little information regarding cats as reservoirs for zoonotic agents, owners and animal handlers are at risk of infection with some viruses, bacteria, fungi and numerous endo- and ectoparasites. However, misinformation is common, e.g. overestimation of the risk of cat ownership for toxoplasmosis [26]. Veterinary team professionals therefore must be well informed about zoonotic agents associated with cats and be able to communicate such information effectively to cat owners, particularly about routes, modes of transmission and prevention. Transmission of zoonotic pathogens may occur through feces (either by direct contact or indirectly through contaminated soil, water or raw produce); hair (e.g., dermatophytes, either directly or indirectly); oral, eye, respiratory, skin and urogenital secretions/exudates; bites and scratches; shared environmental exposure (e.g., inhalation of spores for systemic fungi like *Blastomyces dermatitidis*); and shared vectors such as fleas, ticks, mites, sand flies, mosquitoes or flies (see Table 1). The intensive self-grooming of cats increases the risk for the potential spread of pathogens from anal, genital and ocular mucous membranes to the mouth of the cat and further spread with saliva to the fur [27–29].

Scratches and bites are common transmission modes for zoonotic pathogens. Indeed, an average of 1% of all emergency room visits (per year) in the US are to evaluate people bitten by animals [30], and an estimated 400,000 cat bites and 4.5 million dog bites occur in the US every year [31]. Other high-income countries such as Australia, Canada and France have comparable annual incidence rates for dog bites; worldwide cat bites account for 2–50% of injuries related to animal bites [31], of which a large percentage become infected. The risk of human infection from scratches and bites is increased when the cat owner/handler is immunocompromised and/or the cat they are in contact with is showing clinical signs of a disease [7]. The risk of zoonotic pathogen transmission should be evaluated by the attending veterinarian and discussed with the owner, including appropriate deworming, vector control and handling recommendations.

Zoonotic agents occur across all relevant pathogen types, i.e. parasites, viruses, fungi and bacteria. Regarding parasites, there are a few that need particular zoonotic attention: *Toxoplasma gondii* is still one of the most important zoonotic pathogens. Toxoplasmosis in people is a multisystemic disease that causes granulomatous inflammation in several tissues. Especially at risk are immunocompromised individuals, where infection frequently presents with pulmonary disease or diffuse encephalitis [32], and seronegative, naïve, pregnant women, as intrauterine infection causing congenital toxoplasmosis may cause abortion, neonatal death or fetal abnormalities with detrimental consequences for the new-born child [33–36]. Additionally, infection with *T. gondii* has also been recognized as an important cause of retinochoroiditis in humans [37], as a result of either prenatal infection or an infection that was acquired postnatally [38]. Cats are the definitive host of *T. gondii* and typically shed oocysts for only 2 weeks in a cat’s life [39]. Humans can become infected after accidental ingestion of oocysts (which have had time [days] to sporulate, as they are not immediately infective after excretion/shedding) from cat feces, feces-contaminated soil, water, fruits or vegetables, or through the ingestion of raw meat containing tissue cysts. Due to the restricted time of oocyst shedding in cats and the time needed for oocysts to sporulate to reach an infective stage, food-borne sources as well as soil and water contact may be greater human risk factors for toxoplasmosis rather than direct contacts with cats. Thus, while cat ownership is often top of mind when considering risk reduction measures for toxoplasmosis, an awareness of the other described sources of infection for people, which demand different control measures, for example, ensuring meat is well cooked, raw produce is well washed and hands are thoroughly washed following gardening or outdoor play is required [26]. Further potential zoonotic protozoan feline parasites are *Giardia duodenalis* and *Cryptosporidium* spp. *Giardia duodenalis*, also named *Giardia intestinalis* and *Giardia lamblia*, which is the species that infects mammals. *Giardia duodenalis* is considered a species complex that comprises several genotypes or assemblages; assemblages A and B frequently infect humans and other mammals, while others are host-specific [40]. Most *G. duodenalis* isolates from cats are typed as assemblage F [40, 41]. However, besides this cat-specific assemblage, cats harbor other genotypes that can be transmitted to humans such as assemblages A and B [40]. Regarding *Cryptosporidium* spp., cats are rarely infected, and typically with host-specific *Cryptosporidium felis*, while humans are usually infected with *Cryptosporidium hominis* and *Cryptosporidium parvum* [42]. However, *C. felis* infections have also been detected in humans [43]. People generally acquire *Cryptosporidium* spp. and *G. duodenalis* infections by drinking contaminated water during recreation or by direct contact with infected cattle. *Cryptosporidium felis* oocysts and *G. duodenalis* cysts are immediately infective after excretion/shedding, so people with immunosuppression should be careful when handling cats with diarrhea.

Vector-borne zoonotic protozoans include the agents of some severe human diseases such as zoonotic leishmaniosis, caused by *Leishmania infantum* and transmitted by phlebotomine sand fly bites in both the Old and New Worlds, and American trypanosomosis or Chagas disease, caused by *Trypanosoma cruzi* and transmitted by contamination with triatomine bug feces. Other *Trypanosoma* spp. such as *Trypanosoma evansi* (primarily transmitted by biting flies), *Trypanosoma brucei brucei* and *Trypanosoma congolense* (both mainly transmitted by tsetse flies) have been reported in cats with clinical signs, but to a lesser extent than in dogs. These three protozoans, common in many animal species, have been very rarely associated with atypical human infections [44]. While vector-borne, for all these protozoans an additional risk of mechanical infection exists, especially via direct blood contact (e.g. needle injury when collecting a blood sample).

*Toxocara cati*, *Toxocara malaysiensis* (in Southeast Asia) and hookworms (*Ancylostoma braziliense, Ancylostoma tubaeforme, Ancylostoma ceylanicum* and *Uncinaria stenocephala*) are common helminthic endoparasites of cats with proven or suspected zoonotic potential. *Toxocara cati* can cause visceral, ocular and neural larva migrans in humans, with children at relatively higher risk of *T. cati* infection due to behavioral predilections like geophagia, pica and coprophagia. Hookworm, in particular *A. braziliense*, is the most common cause of ‘creeping eruptions’ or chronic cutaneous larva migrans in people [45]. *Ancylostoma ceylanicum* is an important emerging zoonosis and now recognized as the second most common hookworm infecting humans in the Asia Pacific [46, 47]. *Echinococcus multilocularis* is another relevant zoonotic endoparasite causing severe alveolar echinococcosis in people. Cats can serve as a definitive host for *E. multilocularis*, but their role in maintaining the life cycle and their true zoonotic risk is under debate [48, 49]. However, cats have been shown as a possible source of infection for humans in Europe, though to a lesser extent than dogs [50, 51]. Cats become infected with *Dipylidium caninum* by ingestion of infected fleas or lice when grooming [52]. Humans, particularly young children, can acquire *D. caninum* infection by accidental ingestion of infected fleas. *Dirofilaria* spp. and *Brugia* spp. are zoonotic filarial nematodes transmitted by mosquito bites to cats in endemic areas. Human lymphatic filariosis caused by *Brugia malayi* occurs in South India, Sri Lanka and some foci in Southeast Asia. In endemic areas of Europe and the Americas, *Dirofilaria immitis* is mainly responsible for unifocal or multifocal pulmonary nodules in people. *Dirofilaria repens* is distributed in Europe and in Asia and may cause a wide range of symptoms in people based on the tissue involved during migration and the location of the final subcutaneous nodular lesions [53]. Human thelaziosis caused by the two feline eyeworm species, *Thelazia callipaeda* and *Thelazia californiensis*, may manifest as conjunctivitis, but keratitis and corneal ulcers can also occur [54–56].

To avoid oral infections with parasites, cats should be prevented from hunting and ingesting raw meat, as infected intermediate hosts or contaminated raw meat may represent sources of infection for endoparasites. Daily removal of feces from the soil or litter tray will decrease the risk of environmental contamination and infection in animals and humans by *T. gondii* oocysts, hookworm and *Toxocara* eggs as they are not immediately infective after being excreted in cat feces. This is in contrast to *E. multilocularis* eggs (morphologically indistinguishable from *Taenia* spp. eggs), which are immediately infective, thus demanding feces removal with special care.

For all vector-transmitted parasites, comprehensive vector control is the most relevant approach. For some parasites, e.g. heartworm, specific prophylaxis is essential [57–60].

While several viral infections occur in cats (see above), only a few viruses, such as rabies virus and several other lyssaviruses, are recognized as potential agents of viral zoonoses. Several cases of cat-associated zoonotic cowpox infections, leading to dermal subcutaneous tissue necrosis, neurogenic inflammation, colliquative lymphadenitis or ocular disease, have been described in people exposed to cat scratches or bites [61–65]. In fact, it is estimated that 50% of human cowpox cases in the UK are due to transmission from cats [66]. Other well-recognized viruses, such as the avian influenza virus H7N2, can also be transmitted from cats to humans [67–72]. Vector-borne viruses infecting both cats and humans include West Nile virus and, in Asia, *Dabie bandavirus*, a phlebovirus causing the severe fever with thrombocytopenia syndrome (SFTS) (see Table 1).

*Campylobacter*, *Salmonella*, *Clostridium* and *Yersinia* are a few examples of enteric zoonotic bacteria that can be passed to humans by feces (i.e. ingestion of the infectious agent in contaminated food, water, other environmental sources or via hands) or direct contact with infected cats. Additionally, enteropathogenic *Escherichia coli* (EPEC) and enterohemorrhagic *Escherichia coli* (EHEC) are reported in cats, presenting a potential source of human infection [73, 74]. Shiga toxin-producing *Escherichia coli* (STEC), responsible for the hemolytic uremic syndrome in humans, have also been detected in dog as well as in cat fecal samples with virulence genes in common with isolates from humans, thus constituting a potential additional source of human infection [75].

Bacteria of the genera *Bartonella*, *Capnocytophaga*, *Francisella*, *Pasteurella*, *Rickettsia, Staphylococcus* and even *Yersinia* (i.e. *Yersinia pestis*) are known zoonotic pathogens of cats that can cause serious skin and systemic infections in people and that may result in severe sequelae including meningitis, endocarditis, septic arthritis, osteoarthritis and septic shock [66, 76–83]. Besides a general risk of transmission via cat bites with these pathogens, there is an additional exposure risk to antimicrobial-resistant (AMR) bacteria, which have been demonstrated in the feline oral cavity [84]. Urogenital (*Coxiella*, *Leptospira*) or ocular and respiratory (*Bordetella* and *Chlamydia*)-associated pathogens are also common. *Coxiella burnetii* can cause reproductive disorders in animals; clinical signs and symptoms in people are variable, including febrile illness, pneumonia, hepatitis and reproductive disorders. While farm animals are considered the main source for zoonotic infections, cats have also been associated with zoonotic infections [85]. Zoonotic transmission can occur by aerosol contamination after contact with placenta or amniotic fluids of both aborting and healthy cats [85, 86]. *Leptospira* spp. can cause infections in mammals, including cats and humans [87]. Infection is transmitted by direct contact with infected urine or by contact with infected water or soil. Although cats have not been considered as a main source of infection for people, specific antibodies against *Leptospira* spp., *Leptospira* spp. DNA as well as a positive bacterial culture in urine and kidneys have been detected in cats [88, 89]. *Bordetella bronchiseptica* causes chronic respiratory infections in cats, dogs and humans [15]. However, zoonotic transmission is infrequent, and most cases occur in immunocompromised patients. *Chlamydia felis* causes respiratory and ocular infections in cats, particularly in multi-cat environments [90]. *Chlamydia felis* is transmitted by direct contact between cats and between humans and cats. Again, the risk of zoonotic transmission is extremely rare and is highest in immunocompromised individuals; infection in humans is mainly asymptomatic or causes acute conjunctivitis [90].

The most common fungi that are directly zoonotic are the dermatophytes (e.g. *Microsporum* spp., *Trichophyton* spp.) and the genus *Sporothrix* spp. Both groups are transmitted from cat to cat and from cat to people [91]. Cats can be subclinical carriers of *Microsporum canis* with long-haired breeds and kittens particularly associated with infected premises. About half of people living in households with dermatophyte-infected cats develop ringworm lesions [92]. In tropical and subtropical regions in Latin America, thermodimorphic fungi of the genus *Sporothrix* are responsible for the most frequent subcutaneous mycosis, with endemic occurrence [93]. Cat-transmitted sporotrichosis caused by *Sporothrix brasiliensis* has been a zoonosis of the south and southeast regions of Brazil for more than 20 years [94]. The systemic fungi *Blastomyces dermatitidis*, *Coccidioides immitis* and *Histoplasma capsulatum*, which are common to some countries like the USA, are acquired from the environment. Thus, outdoor cats in endemic areas are of greatest risk. These agents are generally not transferred among cats or between cats and their owners and handlers [95].

Zoonoses with a preferred direction of transmission from humans to animals should also be considered. For example, cats have been shown to be susceptible to SARS-CoV-2 infection acquired in COVID-19-positive households with mild to severe feline respiratory disease observed [96, 97]. However, cats shed SARS-CoV-2 for only short periods of time, and to date only one case of cat-to-human transmission has been reported [98, 99]. Other examples for this direction of transmission include influenza A viruses, *Mycobacterium tuberculosis*, methicillin-resistant *Staphylococcus aureus* (MRSA), *Helicobacter pylori*, *Entamoeba histolytica*, *Streptococcus pneumoniae* and other *Streptococcus* spp.

Many of the above-mentioned zoonotic pathogens are vector-borne (see Table 1). Up to 80% of *Ctenocephalides felis* collected from cats contain the DNA of either a human or cat pathogen [100], with *Bartonella* spp. like *Bartonella henselae* and *Bartonella clarridgeiae*, *Rickettsia felis* and the hemoplasmas being the most common. Thus, any type of cat with increased risk of flea infestation is more likely to be a carrier of human or feline pathogens. Tick-borne disease agents have also been increasingly found in the blood of cats as molecular diagnostic techniques have become more sensitive and available to veterinary health care providers. We now know that tick-infested cats can harbor several zoonotic as well as non-zoonotic infectious agents, such as *Anaplasma* spp. (both *Anaplasma phagocytophilum* and *Anaplasma platys*), *Bartonella* spp., *Babesia* spp., *Borrelia* spp., *Cytauxzoon* spp., *Ehrlichia* spp., *Hepatozoon* spp. and *Rickettsia* spp. [101–104].

Generally, individual cats can be infected subclinically with feline vector-borne pathogens and therefore may be potentially homed into households or locations previously naïve to the corresponding agent. The clinical signs of disease associated with feline vector-borne pathogens have been reviewed in detail [9, 10, 105]. For the flea- and tick-borne agents, fever is generally the most common clinical sign (see Table 1).

## Screening, testing, education and recommendations

When cats are relocated, disease risks are often underestimated. This is in part due to a lack of data, especially regarding vector-borne pathogens in cats and, as mentioned earlier, because cats are often perceived to be at a lower risk of tick-borne pathogens in particular or arthropod-borne diseases in general. Vector-borne pathogens in cats commonly occur, pose disease risks to the individual cat as well as the wider feline populations and have zoonotic potential (see Table 1). Arthropod infestations on relocated cats pose a particular risk in terms of zoonotic exposure and wider spread of infection. Measures to prevent vector-borne pathogen transmission and establishment of exotic vectors are therefore an important consideration, and veterinary team professionals have a vital role to play in raising awareness among cat owners and rescue charities, maintaining biosecurity, reducing zoonotic risk and improving the welfare of relocated cats. When assessing homing cats, professionals should:

i. *Educate the public* regarding the risks of adopting cats from abroad or distant regions. Education regarding the benefits of local cat adoption and the importance of considering the lifestyle of the cat will also enable potential new owners to make informed choices. This communication should be compassionate and non-confrontational as most charities working in this field, and people adopting pets, do so with the best of intentions.
ii. *Ensure that neutering has been carried out and that the cat ideally has a registered microchip or an alternative form of identification in case microchipping is unavailable.* As well as playing an important role in population control, neutering helps prevent horizontal (venereal and through fighting) and vertical transmission of pathogens. Microchipping or alternative identification allows previous treatment, vaccination status and testing records to be traced.
iii. *Ask about origin and travel history for any recently acquired cat*. This will allow for the selection of appropriate diagnostic tests and potential treatments depending on pathogens and vectors present in the region of origin and clinicopathological alterations. Online maps are available for professionals to consult to determine known risks in different geographical locations (e.g. https://www.esccap.org/guidelines-maps/, https://capcvet.org/maps/#/, https://cvbd.elanco.com/cvbd-maps).
iv. *Perform a thorough clinical examination*. Particular attention should be paid to the oral cavity, eyes, skin, feet and claws/nail beds as these areas are particularly likely to be affected by feline vector-borne diseases. Skin lesions may also indicate the presence of current arthropod infestations. Further information on clinical signs associated with vector-borne and other pathogens can be found at the following websites: https://www.esccap.org, https://capcvet.org, https://www.troccap.com, https://cvbd.elanco.com, http://www.abcdcatsvets.org/.
v. *Thoroughly check the cat for parasites*. Check for fleas, ticks and lice and ensure that preventative treatment against these and other ectoparasitic arthropods is in place if ongoing risk is a concern. If allowed to establish, flea household infestations represent an interface where zoonotic pathogens such as *Bartonella* spp., *Rickettsia* spp. and *Dipylidium caninum* could be transmitted. *Rhipicephalus* spp. and *Ixodes* spp. ticks are also capable of establishing infestations in households, the former indoors and in catteries, and both in gardens, allowing onward transmission of tick-borne pathogens. The importance of ongoing preventative arthropod prevention should be emphasized to cat owners and handlers, if there is a recognized risk, to limit zoonotic risk and vector-borne pathogen transmission. Check for endoparasites and ensure that owners are advised on an effective treatment regime based on origin of cat, diagnosed infections and future exposure risk.
vi. *Ascertain the cat’s FeLV/FIV status and consider clinical pathology evaluations*. As well as being significant pathogens in their own right, FeLV and/or FIV are risk factors for several other infections in cats. Biochemistry, hematology profiles and urinalysis are also useful to check for thrombocytopenia, anemia and hyperglobulinemia or proteinuria, for example, because these can be suggestive of vector-borne diseases.
vii. *Report all relevant findings, especially foreign arthropods and pathogens. *Very few vector-borne pathogens in cats are notifiable by law. Therefore, reporting unusual findings to local health authorities, universities, independent organizations such as those mentioned in point IV and peer-reviewed publications will help generate an up-to-date picture of where vectors and pathogens may be emerging. Examples of published reports include *Leishmania* spp. and *Hepatozoon* spp. found in cats in Germany [106], *Rhipicephalus pusillus* found on cats in France [107] or *Haemaphysalis leachi* found on cats imported from Africa to the UK [108].

Cooperation with cat charities and rescue organizations is beneficial as the above steps are most effective if carried out before homing takes place. Adequate arthropod protection, before homing or during the TNR program, will minimize the risk of flea-, tick-, mosquito- or sand fly-borne pathogen transmission. Identification of infection and disease before relocation and including this evaluation in any viability assessment for homing will also help to reduce further transmission of any existing infections and help charities and potential new owners to assess the long-term disease risks for any individual cat.

## Consensus statement

Large numbers of unowned cats continue to constitute an animal welfare, ecological, societal and public health problem worldwide. While well-planned and executed population management programs, such as Trap-Neuter-Return (TNR), are a key component in the long-term control of street cat numbers, other strategies are required for managing existing populations. Relocation and homing of unowned cats is one strategy used in many parts of the world. However, a lack of understanding of an individual cat’s lifestyle and disease status can lead to animal and/or owner stress, the dissemination of feline pathogens and an increased risk of exposure to zoonotic agents. Raising awareness of these issues among veterinary team professionals and those working with cat charities is therefore essential. This includes knowledge of the cat’s lifestyle, including how this influences exposure to pathogens, the geographic distribution of cat pathogens, their clinical signs and/or clinicopathological abnormalities, vectors, modes of transmission, vector and parasite control and evaluation of the potential zoonotic risks. Appropriate testing, surveillance, recording and reporting of infectious agents in homed cats is also a vital component in tracking the geographic spread and emergence of feline pathogens and zoonoses.

## Conclusions

While large numbers of unowned cats continue to be a welfare issue globally, a multifaceted approach to controlling cat numbers and associated pathogen transmission is vital. The increased relocation and homing of unowned cats to reduce feline suffering and social problems mean that strategies are required to reduce accompanying pathogen spread and zoonotic risk. Increasing veterinary education regarding cat lifestyles, at risk pathogens and their vectors alongside increased testing, surveillance and overpopulation control with TNR systems is key to achieving these aims.

## Data Availability

Not applicable.

## Footnotes

1 The term homing is used rather than rehoming in recognition that not all cats in need of homing have previously had homes as pets and because the appropriate solution for some cats is to provide an alternative lifestyle outdoors for them rather than homing them as a pet.
